# Hepatic vein migration of a totally implantable venous access port-a-cath for chemotherapy in a breast carcinoma patient: case report

**DOI:** 10.1590/1677-5449.202101891

**Published:** 2022-05-09

**Authors:** Augusto Cesar Maia Rio Lima Silveira, Paula Shelda Fonseca Fernandes, Danilo Rafael da Silva Fontinele, Rafael Everton Assunção Ribeiro da Costa, José Eduardo Prado Araújo, Wilson de Oliveira Sousa, Sabas Carlos Vieira

**Affiliations:** 1 Centro Universitário Uninovafapi, Teresina, PI, Brasil.; 2 Universidade Estadual do Piauí – UESPI, Teresina, PI, Brasil.; 3 Hospital de Terapia Intensiva – HTI, Teresina, PI, Brasil.; 4 Universidade Federal do Piauí – UFPI, Teresina, PI, Brasil.; 5 Universidade Estadual de Campinas – UNICAMP, Campinas, SP, Brasil.; 6 Oncocenter, Teresina, PI, Brasil.

**Keywords:** complications, catheters, neoadjuvant chemotherapy, hepatic veins, breast neoplasms, case reports

## Abstract

A totally implantable venous access port (TIVAP) is used for chemotherapy administration. Venous port migration to the systemic circulation occurs in less than 1% of complications. The aim of this study is to describe a case of TIVAP migration to the hepatic vein. A 44-year-old female patient with breast cancer was prescribed neoadjuvant chemotherapy. A port-a-cath was surgically implanted for chemotherapy. During the port puncture procedure, blood returned normally when aspirated. When the port was first accessed and flushed with saline solution, swelling was observed at the port site and blood could no longer be aspirated. A chest radiography showed catheter embolization in the region of the hepatic vein. The catheter was retrieved using a snare technique (without complications) and the patient was discharged the next day. The care team should be alert to possible TIIVAP malfunction.

## INTRODUCTION

Totally implantable venous access ports (TIVAP) are commonly indicated for patients who need long-duration chemotherapy for cancer treatment. Migration of a chemotherapy TIVAP catheter into the systemic circulation is a rare event, accounting for less than 1% of all complications related to these devices. Embolization of fragments may occur in the atrium, ventricle, pulmonary artery, hepatic veins, or vena cava, among other sites.^1^^,^^2^

The objective of this study is to describe a case of migration of a chemotherapy TIVAP catheter to the hepatic vein.

## CASE DESCRIPTION

A 44-year-old female patient was referred to the service with a breast nodule. Results of anatomopathological and immunohistochemical tests of a biopsy of the nodule indicated that it was an invasive breast carcinoma of no special type (IBCNST), G2, nuclear grade 2, with estrogen receptors (ER)+: 80%, progesterone receptors (PR)+: 40%, human epidermal growth factor receptor 2 (HER2) negative: 0-1, and Ki67: 30%.

**Figure 1:**
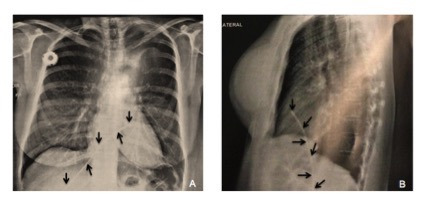
**(A)** and **(B)** Chest X-ray showing migration of the chemotherapy totally implantable venous access catheter to the hepatic vein. **Explanatory Note:** Arrows indicate that totally implantable venous catheter for chemotherapy migrated to hepatic vein.

The patient was prescribed neoadjuvant chemotherapy, and a port-a-cath catheter was placed by phlebotomy with access via the right cephalic vein. The position of the catheter in the superior vena cava was confirmed intraoperatively with radioscopy and the catheter was heparinized. The procedure was uneventful, with no complications, and the patient was discharged with no complaints. When the time came to administer the first chemotherapy cycle, the port was accessed and blood returned as normal, proceeding to infusion of saline, at which point swelling was observed at the port site. The chemotherapy procedure was aborted and chest X-rays (Figures 1A and 1B) showed embolization of the catheter in the region of the hepatic vein. The patient did not have any symptoms.

**Figure 2:**
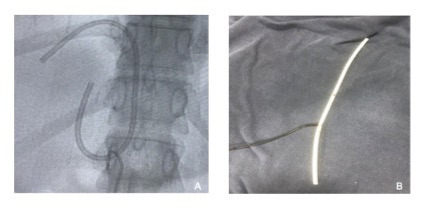
Illustration of the snare technique employed to retrieve the chemotherapy totally implantable venous access catheter migrated to the hepatic vein (using access via the left femoral vein). **(A)** Capturing the catheter; **(B)** Catheter removed.^3^

**Figure 3:**
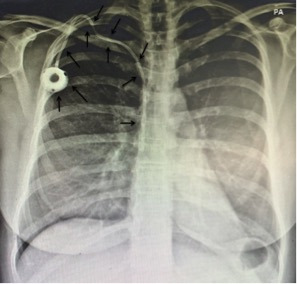
Chest X-ray taken after the procedure to remove the chemotherapy totally implantable venous access catheter that had migrated to the hepatic vein, showing the new port-a-cath fitted without complications. **Explanatory Note:** Arrows indicate that the new venous access port was implanted without complications; The posteroanterior (PA) chest view refers to the X-ray beams passing through the patient from posterior to anterior, with better evaluation of the structures closer to the ventral region.

The patient was admitted and transported to the surgical center for removal of the catheter using a loop snare technique, which was conducted with percutaneous venous access obtained via the left femoral vein. An introducer was inserted to enable manipulation of the materials needed. A catheter with the appropriate curvature to reach the site of the foreign body was advanced over a guidewire and placed adjacent to one of the extremities of the migrated catheter. After the guidewire had been placed, the first catheter was removed and a foreign body retrieval snare catheter was inserted over the same guidewire, positioning it exactly at the extremity of the migrated catheter. At this point, the intravenous foreign body (TIVAP) was captured with the snare (Figure 2A) and withdrawn via the introducer (Figure 2B).^3^ The procedure was conducted with no complications and the patient was discharged with no intercurrent conditions on the first postoperative day. The following month, she underwent placement of a new port-a-cath to proceed with neoadjuvant chemotherapy (Figure 3). This procedure was conducted with no complications. Currently, at around 25-months’ follow-up (December 31, 2019 to January 28, 2022), the patient is in good health and there is no evidence of any complications resulting from migration of the TIVAP or from the retrieval procedure.

This study is part of a larger project with cancer patients seen at a private oncology service in the town of Teresina (Piauí [PI]), Brazil. The project was approved by the research ethics committee at the Universidade Estadual do Piauí, Teresina (PI), Brazil – decision number: 4.311.835 (CAAE: 30154720.0.0000.5209). The study complies with all applicable Brazilian (National Health Council resolution 466/12) and international research ethical principles. The patient signed a free and informed consent form.

## DISCUSSION

The TIVAP is a long-stay device that facilitates infusion of medications, solutions, and chemotherapy agents, blood transfusions, and blood draws for tests. One of its most common indications is for cancer patients who require long duration chemotherapy.^1^^,^^4^ This type of catheter reduces the risk of leakage of irritant chemotherapy substances, offers continuous access, and avoids the discomfort of multiple vein punctures.^4^^,^^5^

It is important to emphasize that, despite the rarity of their occurrence, using a TIVAP involves the potential for complications, such as infection, embolization, catheter occlusion, venous perforation, atrial perforation, arrhythmia, phlebitis, leakage, migration, and catheter fracture, among others.^5^^-^8 These complications can be classified as early complications (within 7 days of implantation) or late complications (after this period).^4^

Voog et al.^9^ published a prospective, single-center, observational study with 483 patients who had undergone implantation of at least one TIVAP between January 1, 2006 and December 31, 2006, with a total of 493 TIVAPs fitted, equating to a total of 367,359 catheter-days. These patients were followed until removal of the device, death, or end of follow-up on December 31, 2013. Over a mean follow-up of 18 months (1–94 months), the study reported 87 complications, described in Table 1 (0.237 complications/1,000 catheter-days). The three most common complications were infections (37 cases), thrombotic events (17 cases), and extravasations (9 cases).

**Table 1:** Possible complications caused by totally implantable venous access ports (TIVAP) observed in a study by Voog et al.^9^ with 483 patients with TIVAP fitted.

**Complications**			**N (%)**
Infections			37 (42.5)
Thromboses			17 (19.5)
Extravasations			9 (10.3)
Port seal defects			6 (6.9)
TIVAP separation			3 (3.4)
Catheter rupture			2 (2.3)
Pocket hematoma			1 (1.1)
Pneumothorax			1 (1.1)
TIVAP migration			1 (1.1)
Non-functional TIVAP			1 (1.1)
Wound dehiscence			1 (1.1)
Pain			1 (1.1)
TIVAP externalization			1 (1.1)
Inflammation of TIVAP pocket without documented infection			1 (1.1)
Others			5 (5.7)
		**Total**	**87 (100.0)**

In the present study, the patient suffered an early complication involving embolization of the catheter – a rare event that is reported in just 1% of TIVAP complications.^1^^,^^3^ Migration may involve the entire length of the catheter or part of it, in cases of catheter fracture. In the present case, the entire catheter had migrated; and the catheter had therefore become disconnected from the port, which could occur because of excessive pressure during infusion of drugs or because of poor surgical technique. In contrast, fracture is generally a late complication and may be linked with catheters placed by puncture, resulting from compression of the catheter where it passes between the clavicle and the first rib. The rate of TIVAP fracture is lower when the catheter is fitted using dissection, whether of the cephalic vein or the external jugular.^1^

Embolization may occur in the atrium, ventricle, pulmonary artery, hepatic veins, or vena cava and, because of circulation of fragments, the major clinical concerns are occurrence of cardiac perforation, arrhythmia, sepsis, and pulmonary emboli. Despite this, patients with embolization have scant symptomology and are generally asymptomatic, so diagnostic suspicion is aroused when it is impossible to perform infusion or reflux of blood is absent.^1^ In the case described here, the condition was suspected immediately before administration of chemotherapy, when swelling around the port site was observed and no blood could be aspirated.

Death from embolization by chemotherapy TIVAP is rare, and diagnosis is achieved by radiography showing the foreign body. Treatment must always be provided promptly, to avoid major complications, and the endovascular technique is standard treatment, as performed in the patient described.^1^ In the past, surgery was the only treatment option for fractured and migrated catheters. However, percutaneous removal of migrated catheters is linked with lower morbidity and mortality than surgical procedures.^5^

## CONCLUSIONS

Migration of TIVAP fragments to the hepatic vein is an extremely rare and potentially lethal complication. Treatment must therefore be provided as early as possible and the standard treatment employs the endovascular access technique because of its low rate of complications. Despite the rarity of events such as these, the chemotherapy team must always be alert to any type of difficulty with drawing blood or administrating liquid via the catheter.
