# Supplementary material for: Hepatic vein migration of a totally implantable venous access port-a-cath for chemotherapy in a breast carcinoma patient: case report
Source: J Vasc Bras. 2022 May 9;21:e20210189. doi: 10.1590/1677-5449.202101891 (PMC9083582; doi:10.1590/1677-5449.202101891)
Supplement: Translation

# Translation: Migração para veia hepática de cateter totalmente implantável para quimioterapia em paciente com carcinoma de mama: relato de caso

**DOI:** 10.1590/1677-5449.202101892

**Authors:** Augusto Cesar Maia Rio Lima Silveira, Paula Shelda Fonseca Fernandes, Danilo Rafael da Silva Fontinele, Rafael Everton Assunção Ribeiro da Costa, José Eduardo Prado Araújo, Wilson de Oliveira Sousa, Sabas Carlos Vieira

**Affiliations:** 1 Centro Universitário Uninovafapi, Teresina, PI, Brasil.; 2 Universidade Estadual do Piauí – UESPI, Teresina, PI, Brasil.; 3 Hospital de Terapia Intensiva – HTI, Teresina, PI, Brasil.; 4 Universidade Federal do Piauí – UFPI, Teresina, PI, Brasil.; 5 Universidade Estadual de Campinas – UNICAMP, Campinas, SP, Brasil.; 6 Oncocenter, Teresina, PI, Brasil.

**Keywords:** complicações, cateteres, quimioterapia neoadjuvante, veias hepáticas, neoplasias da mama, relatos de caso

## Abstract

O cateter totalmente implantável (CTI) é utilizado na administração da quimioterapia. Em menos de 1% dos casos de complicação, pode ocorrer migração do CTI para quimioterapia para a circulação sistêmica. O objetivo deste estudo foi descrever um caso de migração do CTI para a veia hepática. Uma paciente do sexo feminino, de 44 anos de idade, teve diagnóstico de câncer de mama com indicação de quimioterapia neoadjuvante. Realizou-se a implantação de cateter *port-a-cath*. Durante o procedimento de punção do cateter, houve retorno normal de sangue, e foi realizada infusão de soro fisiológico. Em seguida, houve um aumento de volume no local do *port* e não retorno de sangue à aspiração. A radiografia de tórax mostrou embolização do cateter em topografia hepática. Retirou-se o cateter pela técnica do laço (sem complicações), e a paciente recebeu alta no dia seguinte. Possíveis alterações no funcionamento do CTI devem chamar atenção da equipe responsável.

## INTRODUÇÃO

O uso de cateter totalmente implantável (CTI) é comumente indicado para pacientes que necessitam de quimioterapia de longa duração no tratamento do câncer. A migração do CTI para quimioterapia para a circulação sistêmica é um evento raro e representa menos de 1% das complicações relacionadas a esse dispositivo. A embolização do fragmento pode ocorrer para átrio, ventrículo, artéria pulmonar, veias hepáticas e veia cava, entre outros^1^^,^^2^.

O objetivo deste estudo foi descrever um caso de migração do CTI para quimioterapia para a veia hepática.

## DESCRIÇÃO DO CASO

Uma paciente do sexo feminino, de 44 anos de idade, chegou ao serviço apresentando nódulo mamário. Os resultados da biópsia do nódulo com estudos anatomopatológico e imuno-histoquímico indicaram se tratar de um carcinoma invasivo de mama de tipo não especial (CINE), G2, grau nuclear 2, receptores de estrogênio (RE)+: 80%, receptores de progesterona (RP)+: 40%, receptores do fator de crescimento epidérmico humano 2 (HER2) negativos: 0-1 e Ki67: 30%.

A paciente teve indicação de quimioterapia neoadjuvante, e foi realizada implantação de cateter *port-a-cath* com acesso pela veia cefálica direita por flebotomia. A posição do cateter na veia cava superior foi confirmada por radioscopia no intraoperatório, e, em seguida, procedeu-se a heparinização do cateter. O procedimento ocorreu sem intercorrências, e a paciente recebeu alta sem queixas. No momento da administração do primeiro ciclo de quimioterapia, houve retorno de sangue após punção do cateter, iniciando-se, em seguida, a infusão de soro fisiológico, sendo, então, observada elevação no sítio do reservatório do cateter (*port*). O procedimento de quimioterapia foi suspenso, e realizou-se uma radiografia de tórax (Figuras 1A e 1B) que mostrou embolização do cateter em topografia hepática. A paciente não apresentou nenhum sintoma.

**Figura 1:** **(A)** e **(B)** Radiografia de tórax mostrando migração de cateter totalmente implantável para quimioterapia para a veia hepática. **Nota explicativa:** As setas apontam o cateter totalmente implantável para quimioterapia migrado para veia hepática.

A paciente foi internada e encaminhada para o centro cirúrgico para retirada do cateter pela técnica do laço, que foi realizada através de acesso venoso percutâneo pela veia femoral esquerda. Realizou-se o implante de um introdutor, permitindo o manejo dos materiais a serem utilizados. Um cateter com curvatura apropriada para atingir a localização do corpo estranho foi conduzido sobre fio guia e posicionado adjacente a uma das extremidades do cateter. Após o posicionamento do fio guia, retirou-se o cateter e introduziu-se sobre o mesmo fio guia o dispositivo de extração do corpo estranho (laço), até posicioná-lo exatamente na extremidade do cateter localizada anteriormente. Neste momento, o corpo estranho intravenoso (CTI) foi capturado pelo dispositivo (Figura 2A) e conduzido até o introdutor para a retirada (Figura 2B)^3^. O procedimento ocorreu sem complicações, e a paciente recebeu alta sem intercorrências no primeiro dia de pós-operatório. No mês seguinte, a paciente foi submetida à implantação de um novo cateter *port-a-cath* para a realização da quimioterapia neoadjuvante (Figura 3). O procedimento ocorreu sem complicações. Atualmente, com cerca de 25 meses de seguimento (31/12/2019 a 28/01/2022), a paciente se encontra bem e sem evidências de complicações decorrentes da migração do CTI ou do procedimento de retirada.

**Figura 2:** Ilustração da técnica do laço realizada para retirada de cateter totalmente implantável (CTI) para quimioterapia com migração para a veia hepática (acesso através da veia femoral esquerda). **(A)** Captura do CTI; **(B)** CTI retirado^3^.

**Figura 3:** Radiografia de tórax realizada após o procedimento de retirada do cateter totalmente implantável para quimioterapia com migração para a veia hepática mostrando o novo cateter *port-a-cath* implantado sem complicações. **Nota explicativa:** As setas apontam o novo cateter port-a-cath implantado sem complicações; No contexto de uma radiografia, a sigla PA (póstero-anterior) refere-se à direção do raio-X atravessando o paciente de posterior para anterior, avaliando melhor estruturas mais próximas da região ventral.

Este estudo faz parte de um projeto em pacientes com câncer atendidos em um serviço privado de Oncologia localizado na cidade de Teresina (Piauí [PI]), Brasil. O projeto foi aprovado pelo Comitê de Ética em Pesquisa da Universidade Estadual do Piauí, Teresina (PI), Brasil – número do parecer: 4.311.835 (CAAE: 30154720.0.0000.5209). Foram respeitados todos os preceitos éticos em pesquisa vigentes no Brasil (resolução do Conselho Nacional de Saúde número 466/12) e nos documentos internacionais. A paciente forneceu termo de consentimento livre e esclarecido.

## DISCUSSÃO

O CTI é um dispositivo de longa permanência que auxilia a infusão de medicamentos, soluções e quimioterápicos, transfusões sanguíneas e coleta de exames. Entre suas principais indicações, estão os pacientes oncológicos que necessitam de quimioterapia de longa duração^1^^,^^4^. O uso desse tipo de cateter reduz o risco de extravasamento de substâncias quimioterápicas irritantes, além de possibilitar um acesso contínuo e evitar o desconforto do paciente em relação às numerosas venopunções^4^^,^^5^.

É importante salientar que, apesar de ocorrer raramente, o uso de CTI tem o potencial de causar complicações ao paciente, como infecções, embolizações, oclusões do cateter, perfurações venosas, perfurações atriais, arritmias, flebites, vazamentos, migrações e quebras do cateter, entre outras^5^^-^8. Desse modo, elas podem ser classificadas como complicações precoces (até 7 dias após a implantação) ou tardias, posteriormente a esse período^4^.

Voog et al.^9^ realizaram um estudo observacional prospectivo em um único centro com 483 pacientes que haviam sido submetidos à implantação de pelo menos um CTI entre 01/01/2006 e 31/12/2006 em um total de 493 CTIs implantados, representando uma quantidade global de 367,359 cateteres-dia. Esses pacientes foram acompanhados até a data de remoção do dispositivo, óbito ou término do seguimento (31/12/2013). Em um seguimento médio de 18 meses (1–94 meses), esse estudo mostrou a ocorrência de 87 complicações, descritas na Tabela 1 (0,237 complicações/1.000 cateteres-dia). As três complicações mais comuns foram infecções (37 casos), eventos trombóticos (17 casos) e extravasamentos (9 casos).

**Tabela 1:** Possíveis complicações decorrentes do uso de cateter totalmente implantável (CTI) ocorridas no estudo conduzido por Voog et al.^9^ em 483 pacientes com CTI implantado.

**Complicações**			**N (%)**
Infecções			37 (42,5)
Tromboses			17 (19,5)
Extravasamentos			9 (10,3)
Defeitos no invólucro do *port*			6 (6,9)
Separações do CTI			3 (3,4)
Rupturas do cateter			2 (2,3)
Hematoma cavitário			1 (1,1)
Pneumotórax			1 (1,1)
Migração do CTI			1 (1,1)
CTI não funcional			1 (1,1)
Desunião da cicatriz			1 (1,1)
Dor			1 (1,1)
Externalização do CTI			1 (1,1)
Inflamação da cavidade do CTI sem infecção documentada			1 (1,1)
Outras			5 (5,7)
		**Total**	**87 (100,0)**

Neste estudo, o caso apresentou uma complicação precoce caracterizada pela embolização do cateter – evento raro que está relacionado com apenas cerca de 1% das complicações por CTI^1^^,^^3^. A migração pode ocorrer por todo o trajeto ou por parte dele nos casos de fratura. No presente caso, ocorreu migração de todo o cateter; portanto, houve desconexão entre o *port* e o cateter, o que pode ocorrer por excessiva pressão na infusão de drogas ou por má técnica cirúrgica. Já a fratura geralmente é uma complicação tardia e pode estar associada ao cateter que é colocado por punção, ocorrendo pela compressão do cateter na passagem entre a clavícula e a primeira costela. Assim, quando realizado por dissecção, seja da veia cefálica ou jugular externa, o CTI apresenta uma taxa de fratura menor^1^.

A embolização pode ocorrer em átrio, ventrículo, artéria pulmonar, veias hepáticas e cava e, devido à circulação desses fragmentos, têm-se como principais preocupações clínicas a ocorrência de perfurações cardíacas, arritmias, sepse e embolias pulmonares. Apesar disso, a sintomatologia do paciente com embolização é escassa, geralmente assintomática, tendo-se a suspeita diagnóstica quando há impossibilidade de infusão ou quando o refluxo de sangue é inexistente^1^. No caso descrito, a suspeita aconteceu no momento imediatamente antes da administração da quimioterapia, quando ocorreu aumento do volume no local do *port* e não houve mais retorno de sangue à aspiração.

O óbito por embolização de CTI para quimioterapia é raro, e o diagnóstico se dá pela realização de radiografia para visualização do corpo estranho. Além disso, o tratamento deve ser realizado de forma precoce, buscando evitar maiores complicações, sendo a técnica por acesso endovascular o tratamento padrão, como realizado na paciente do caso descrito^1^. No passado, a cirurgia era a única opção no tratamento de cateteres fraturados e migrados. Porém, a remoção percutânea de cateteres migrados é relacionada a menor morbimortalidade se comparada aos procedimentos cirúrgicos^5^.

## CONCLUSÃO

A migração para veia hepática de fragmentos do CTI é uma complicação extremamente rara e potencialmente letal. Portanto, o tratamento deve ser realizado o mais precocemente possível, sendo a técnica de acesso endovascular o tratamento padrão por apresentar baixas taxas de complicação. Apesar da raridade de eventos como esse, a equipe de quimioterapia deve ficar sempre atenta para qualquer dificuldade de coleta de sangue ou administração de líquidos no cateter.
